# Advancements in Hydrogels for Corneal Healing and Tissue Engineering

**DOI:** 10.3390/gels10100662

**Published:** 2024-10-16

**Authors:** Kevin Y. Wu, Shu Yu Qian, Anne Faucher, Simon D. Tran

**Affiliations:** 1 Department of Surgery, Division of Ophthalmology, University of Sherbrooke, Sherbrooke, QC J1G 2E8, Canada; yang.wu@usherbrooke.ca (K.Y.W.);; 2 Faculty of Medicine, University of Sherbrooke, Sherbrooke, QC J1G 2E8, Canada; 3 Faculty of Dental Medicine and Oral Health Sciences, McGill University, Montreal, QC H3A 1G1, Canada

**Keywords:** hydrogels, biomaterial, corneal tissue engineering, stimuli-responsive gels, corneal transplantation, polymers, nanotechnology, hybrid hydrogels, cell encapsulation, corneal pathologies, tissue repair

## Abstract

Hydrogels have garnered significant attention for their versatile applications across various fields, including biomedical engineering. This review delves into the fundamentals of hydrogels, exploring their definition, properties, and classification. Hydrogels, as three-dimensional networks of crosslinked polymers, possess tunable properties such as biocompatibility, mechanical strength, and hydrophilicity, making them ideal for medical applications. Uniquely, this article offers original insights into the application of hydrogels specifically for corneal tissue engineering, bridging a gap in current research. The review further examines the anatomical and functional complexities of the cornea, highlighting the challenges associated with corneal pathologies and the current reliance on donor corneas for transplantation. Considering the global shortage of donor corneas, this review discusses the potential of hydrogel-based materials in corneal tissue engineering. Emphasis is placed on the synthesis processes, including physical and chemical crosslinking, and the integration of bioactive molecules. Stimuli-responsive hydrogels, which react to environmental triggers, are identified as promising tools for drug delivery and tissue repair. Additionally, clinical applications of hydrogels in corneal pathologies are explored, showcasing their efficacy in various trials. Finally, the review addresses the challenges of regulatory approval and the need for further research to fully realize the potential of hydrogels in corneal tissue engineering, offering a promising outlook for future developments in this field.

## 1. Introduction

Hydrogels have gained considerable attention in biomedical research, particularly for their potential in tissue engineering and regenerative medicine [1]. Among their many applications, the use of hydrogels in corneal healing and tissue engineering stands out due to the critical need for alternative solutions to donor cornea shortages [2]. The cornea is frequently subjected to injuries and diseases that can lead to significant and irreversible vision loss. Conventional treatments for severe corneal pathologies often rely on donor corneas, but the global scarcity of suitable donors has created a pressing demand for innovative solutions [2].

This review focuses on the application of hydrogels in corneal healing and tissue engineering, offering a review of their properties, synthesis processes, and clinical applications. Hydrogels, with their high water content, biocompatibility, and tunable mechanical properties, present an ideal scaffold for corneal repair [1,3,4].

Specifically, this review aims to:

1. Examine the fundamental properties of hydrogels that make them suitable for corneal tissue engineering.
2. Analyze various synthesis processes and types of hydrogels, including stimuli-responsive variants, used in corneal applications.
3. Evaluate current preclinical trials involving hydrogel-based corneal therapies.
4. Identify the challenges and limitations to clinical translation.
5. Provide insights and recommendations for future research directions to enhance the viability of hydrogels as alternatives to corneal transplants.

We discuss the fundamentals of hydrogels, emphasizing their relevance to corneal tissue engineering, and explore various types of hydrogels, including stimuli-responsive variants. By evaluating current preclinical and clinical trials and the challenges of integrating hydrogels into medical practice, this review aims to provide a rationale for the continued exploration of hydrogels as a viable alternative to corneal transplants.

## 2. Fundamentals of Hydrogels

In recent decades, hydrogels have received increasing amounts of attention due to its numerous potential applications both inside and outside the medical field. Notably, engineering, agriculture, and wastewater management have all benefitted from incorporating hydrogels in their respective domains [5]. In terms of biomedical applications, researchers have identified several main usages for hydrogels: tissue engineering, wound dressings, and drug delivery systems [1]. Before delving into their diverse applications, it is essential to first define hydrogels, outline their classification, and highlight the key properties that warrant their extensive investigation.

### 2.1. Definition and Properties

Hydrogels are defined as three-dimensional (3D) structural networks composed of crosslinking polymer chains capable of absorbing large volumes of water [6]. This ability to swell and retain water is attributable to the presence of hydrophilic functional groups branching off the gel’s backbone (–OH, –COOH, –CONH–, –NH_2_, etc.) [1]. Additionally, the chemical bonds between polymers provide structural solidity that prevents undesirable disintegration [7]. By definition, hydrogels are biphasic materials that should consist of at least 10% water, either by weight or by volume. Therefore, they have major similarities to natural tissues in terms of elasticity and softness due to this high water content [8]. The percentage of water absorbed in the interstices is dependent upon numerous factors, most importantly the nature of the swelling media, the strength of bonds between crosslinked chains, the degree of crosslinking, and the attached groups’ hydrophilicity [9,10].

Overall, hydrogels possess multiple desirable and adjustable properties, the most notable of which include biocompatibility, biodegradability, mechanical strength, hydrophilicity, absorbency, and viscoelasticity [11]. Importantly, in order to be viable for human use, the properties of such biomaterials must be comparable to those of the tissue it is replacing. Being non-toxic and non-allergenic are minimal requirements allowing the hydrogel to be implanted safely in the human body with low risk of adverse events [12]. Having a desirable rate of degradation would optimally support regenerating tissues that would slowly replace the hydrogel [13]. An appropriate mechanical strength is necessary for structural stability and adequate functioning of the engineered tissue [14]. A hydrogel’s ability to maintain a high level of water content is essential to provide a proper extracellular matrix (ECM) for cell seeding, differentiation, and proliferation [3]. These properties are all tunable via the selection of appropriate constituent components and synthesis processes to best match the characteristics of the mimicked tissue. Subsequent sections provide a more detailed examination of these properties for corneal applications.

### 2.2. Types of Hydrogels

**Figure 1:**
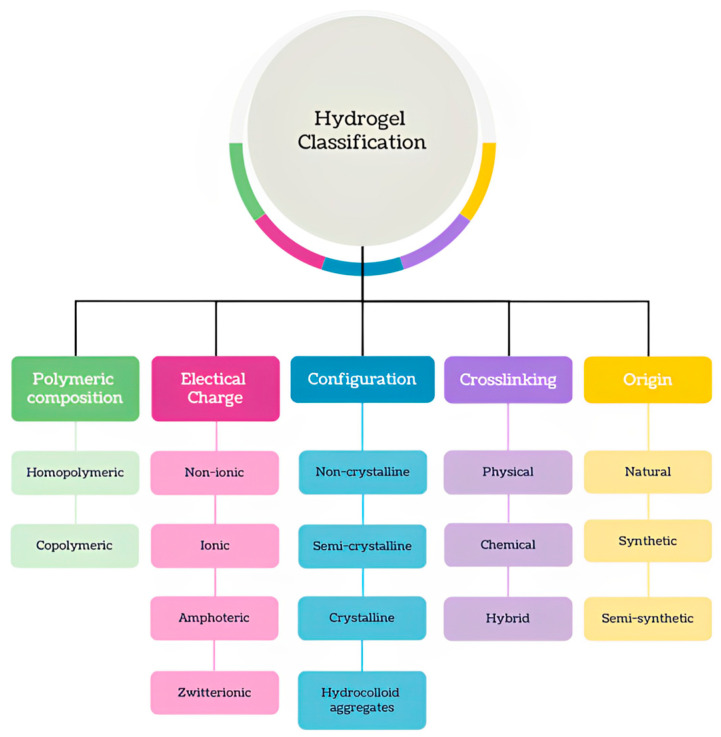
Classification of hydrogels.

There are several methods of classifying hydrogels via different bases. Figure 1 summarizes the different classification systems for hydrogels along with their subgroups. The polymeric composition, electrical charge, configuration, method of crosslinking, and origin are examples of properties by which they can be categorized. Under the basis of electrical charge, gels can be non-ionic, ionic (can be anionic or cationic), amphoteric (containing both basic and acid functional groups), or zwitterionic (made up of repeating units containing equal numbers of anionic and cationic groups for a net charge of zero) [9]. Based on configuration, hydrogels can be placed in one of four categories: non-crystalline, semi-crystalline, crystalline, or hydrocolloid aggregates [15]. Furthermore, hydrogels can be divided depending on the type of crosslinking, whether it is physical, chemical, or a combination of both. Chemical crosslinking consists of covalent bonds, while physical crosslinking involves transient junctions like ionic forces, hydrogen bonds, hydrophobic interactions, or polymer chain entanglements [16,17]. There are numerous techniques to create physically crosslinked hydrogels, like charge interactions, freezing–thawing, and crystallization. Chemical crosslinking is frequently induced through redox polymerization, enzyme-enabled crosslinking, Schiff base reactions, the Diels–Alder reaction, and Michael addition [18]. Hydrogels can also differ with regard to their polymeric composition. While homopolymeric gels are made up of a single monomer species, copolymeric gels incorporate two or more different molecules in its structure [19]. Polyethylene glycol is an example of a homopolymer while poly(acrylamide-co-acrylic-acid) would be considered copolymeric [10].

To better explore the functionality of hydrogels, they are commonly sorted by origin and organized into three main groups: natural, synthetic, and hybrid [10]. Natural hydrogels are frequently derived from collagen, chitosan, hyaluronic acid, gelatin, or cellulose, to name a few examples [20]. Their main advantages include their inherent bioactivity, biocompatibility, and biodegradability, on top of originating from renewable sources [21]. However, they are generally limited by their low mechanical strength and resistance [22]. In contrast to natural polymers, synthetic scaffolds like polyethylene glycol (PEG), polyvinyl alcohol (PVA), polyacrylic acid (PAA), and polyvinylidene fluoride (PVDF) frequently possess opposite characteristics [19,23]. They have been observed to be considerably more stable and stronger, and have greater water retention capabilities, but at the cost of biocompatibility [24]. Although certain synthetic substances such as polyacrylamide (PAAM) are non-toxic, their cytocompatibility remains less than that of natural polymers [25]. To get the best of both types, researchers have attempted to combine natural and synthetic polymers, thus creating a hybrid hydrogel possessing the qualities of all its components. PEG conjugated with gelatin, albumin, or fibrinogen are some of the most commonly studied semi-synthetic hydrogels [26]. Their unique composition optimizes cell regeneration and proliferation without compromising its mechanical integrity [27]. The potential of these materials in corneal tissue engineering will be discussed in greater depth in a subsequent section.

## 3. Cornea Anatomy and Repair Mechanisms

### 3.1. Corneal Structure and Function

**Figure 2:**
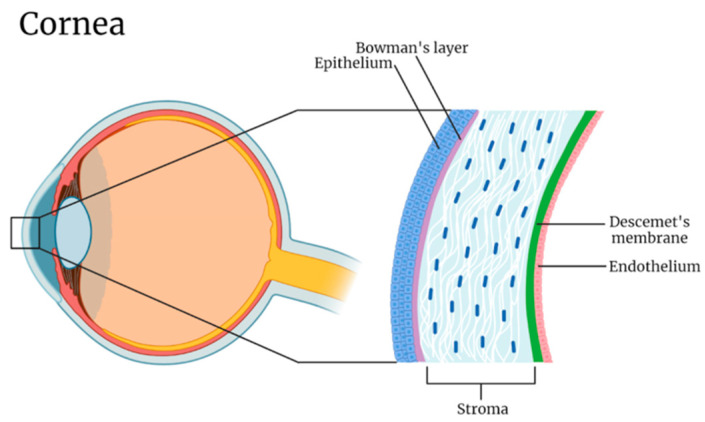
Layers of the cornea. Reprinted from Wu et al., 2024 [32].

The cornea constitutes the transparent outermost covering on the anterior eyeball. Its primary role consists of acting as a protective barrier against pathogens and providing a refractive surface for the eye [28]. Notably, the cornea plays a critical role in vision, contributing approximately 70% of the eye’s total refractive power—around 40 diopters out of the eye’s overall 60 diopters—underscoring its vital importance in one’s visual acuity [29]. Shapewise, a healthy cornea is prolate, convex, and oval [29]. The cornea is thinnest at its center and gradually thickens as it extends toward the periphery, giving it an aspheric surface profile [30]. This tissue consists of both cellular and acellular elements stratified into five distinct layers that, named sequentially from outside to inside, are the epithelium, Bowman’s layer, the stroma, Descemet’s membrane, and the endothelium (Figure 2) [31].

The corneal epithelium, made up of five to seven layers of tightly joined squamous cells, functions as a barrier sheltering inner structures from microbes, blunt trauma, and toxic chemicals [33]. Immediately posterior to the epithelium lies Bowman’s layer, a cell-free collagen and proteoglycan condensate that plays a role in maintaining the cornea’s shape [34]. Continuing inwards, the stroma is the thickest layer of the cornea, accounting for up to 85% of its thickness. It consists of keratocytes residing in an ECM predominantly made of type 1 collagen [35]. These fibers are packed in a parallel fashion, forming fibrils that themselves bundle up into perpendicularly organized lamellae [29,36]. This precise arrangement of collagen fibers and ECM contributes significantly to the cornea’s transparency [36]. Between the stroma and the endothelium lies a dense, acellular matrix containing mostly type 4 collagen and laminin known as Descemet’s membrane [37]. This layer regulates corneal homeostasis by controlling the passage of substances like water and growth factors [38]. Lastly, the corneal endothelium consists of a monolayer of metabolically active cuboidal cells. Lined with high densities of Na-K-ATPase pumps, this structure’s main function lies in its ability to maintain the cornea’s deturgescence [39]. Throughout one’s lifetime, endothelial cell density gradually decreases at an average rate of 0.6% per year, declining from 3500 cells/mm^2^ at birth to about 2500 cells/mm^2^ [40]. The human cornea is highly innervated, yet it is almost devoid of any vascularization. It thus heavily relies on the diffusion of nutrients from the aqueous humor as well as the tear film [41].

### 3.2. Corneal Pathologies and Healing Process

Numerous corneal pathologies exist, ranging from common to rare and from mild to vision-threatening. By etiology, corneal diseases can be categorized into the following groups: infections, inflammation, traumas, systemic metabolic disorders, tumors, dystrophies, degenerations, as well as congenital and developmental anomalies [42]. Globally, from 1990 to 2015, corneal pathologies ranked as the fifth most common cause of blindness [43]. Apart from complete blindness, corneal pathologies are also significant contributors to moderate to severe vision loss [44]. Among them, infectious keratitis is one of the most commonly occurring diseases, and it is responsible for over 1 million medical visits every year in the Unites States alone [45]. Based on the causative organism, it can be classified as bacterial, viral, fungal, or parasitic [46]. If not promptly diagnosed and treated, the pathogen can infiltrate deeper into the stroma, causing thinning and even perforation in the most severe scenario. Such patients would require surgical treatments [47]. Although less prevalent, autoimmune disorders causing corneal ulcers are not to be neglected. There exists an extensive list of differential diagnoses that can mostly be categorized into systemic (e.g., collagen vascular disease, vasculitis, sarcoidosis), dermatological (e.g., pemphigoid, Steven–Johnson syndrome, neutrophilic dermatosis), or unknown, like in the case of Mooren’s ulcer [48]. Sjögren’s syndrome is a significant cause of sicca keratitis, primarily due to chronic inflammation of the lacrimal glands, which reduces tear production [49]. This tear film disruption initiates a vicious cycle, characterized by tear film hyperosmolarity and the release of inflammatory cytokines. These changes compromise the epithelial barrier, potentially leading to corneal melting and perforation if left untreated [50]. Physical and chemical injuries are also well-documented causes of corneal damage. While most cases involve minor injuries, such as small corneal abrasions that can be managed conservatively, more severe situations—such as penetrating trauma and lacerations resulting in an open globe—may require surgical intervention [51]. Degenerative diseases like Fuchs’ dystrophy involves progressive loss of endothelial cells that typically manifests during the later decades of life [52]. As the number of endothelial cells diminishes, this membrane’s ability to regulate the cornea’s hydration balance becomes impaired, with chronic uncontrolled swelling resulting in the formation of ECM excrescences and scar tissue, compromising visual acuity. Treatment often involves managing the corneal edema with hypertonic saline drops or ointments in the early stages, while advanced cases may require surgical interventions such as Descemet’s stripping automated endothelial keratoplasty (DSAEK) or Descemet membrane endothelial keratoplasty (DMEK) to restore corneal endothelial function [53].

**Figure 3:**
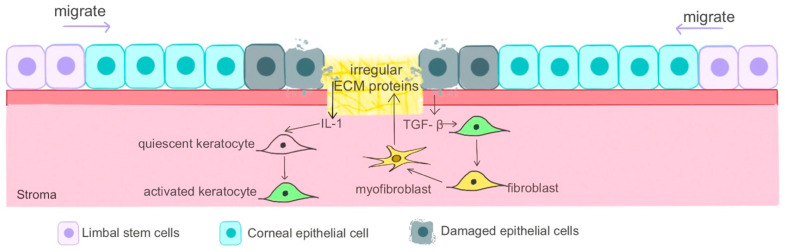
Corneal healing process involving keratocyte activation via interleukins as well as LESC differentiation and migration. Reprinted from Shixu Li et al., 2023 [58]. Used under CC BY-NC-ND 4.0 (https://creativecommons.org/licenses/by-nc-nd/4.0/ (accessed on 30 August 2024)).

The healing process of the cornea is influenced by the extent of the injury as well as the layers affected, with certain layers possessing greater regenerative capacities than others. Immediately following an initial insult, the injured or dead cells release cytokines that bind interleukin receptors expressed by keratocytes in the surrounding stroma [54]. This heightened concentration of cytokines induces apoptosis in these adjacent keratocytes as a possible defense mechanism to prevent viruses from invading deeper into the cornea [55]. Inflammatory chemokines also recruit large numbers of monocytes, lymphocytes, and fibroblasts that in return upregulate the production of growth factors (Figure 3). This increase in growth factors stimulates the activation and proliferation of surviving keratocytes to repair stromal ECM by degrading and replacing disorderly portions of the matrix [56,57].

**Figure 4:**
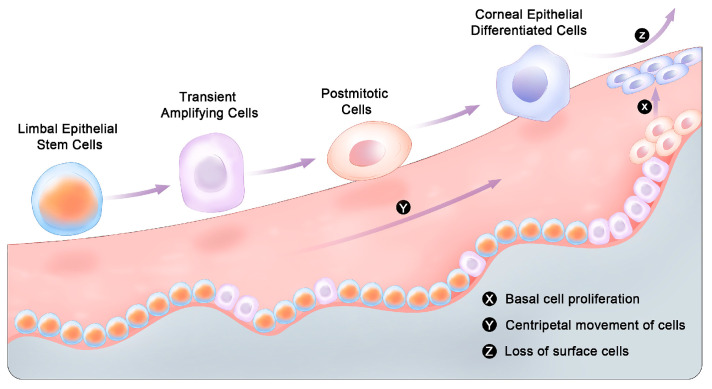
The main steps of corneal cell regeneration. This process speeds up in cases of corneal injuries. Reprinted from Shiding Li et al., 2024 [68]. Used under CC BY-NC-ND 4.0 (https://creativecommons.org/licenses/by-nc-nd/4.0/ (accessed on 30 August 2024)).

Under normal circumstances, the epithelium maintains homeostasis through a process known as the “XYZ” hypothesis (Figure 4). X represents the constant mitotic activity of basal cells; Y, the centripetal movement of peripheral cells; and Z, the desquamation of superficial cells [59]. As a response to injury, the rate of this limbal epithelial stem cell (LESC) division cycle increases 8-fold until wound closure [60]. Congenital or acquired LESC deficiencies or extensive stromal injuries may complicate the healing process and can lead to irreversible fibrosis, neovascularization, and ingrowth of conjunctival cells [61]. In contrast to the epithelial layers, the corneal endothelial cells are halted in the G1 phase and do not have the ability to regenerate [62]. When these cells get destroyed, the remaining ones compensate for this loss by migrating and spreading out evenly over the damaged area [63]. When the endothelial cell density drops below 400 cells/mm^2^, its water regulation capability becomes defective, causing corneal edema and vision compromise [64]. Pathogens like bacteria and viruses can cause deep and extensive damage to the cornea’s epithelium and stroma. In such situations, inflammatory cascades and immune cells recruited may disrupt the normal arrangement of corneal ECM fibers, leading to fibrosis [65]. Hence, hydrogels can be utilized to mitigate this issue by providing a well-organized 3D structure onto which cells and the ECM can regenerate [66]. More broadly speaking, hydrogels also facilitate corneal healing by providing a protective layer over the damaged area that stabilizes that ocular microenvironment. Additionally, hydrogels can be seeded with active molecules or corneal cells to further enhance the healing process [67].

### 3.3. Corneal Transplants

Among eye diseases, not all necessarily lead to complete blindness, and few can be truly considered as vision-threatening. However, ift is well known that eye diseases greatly compromise patients’ quality of life [69]. Vision loss and other debilitating symptoms frequently accompanying eye illnesses are linked to reduced daily activities, social isolation, institutionalization, and the development of mental health disorders [70]. Therefore, proper treatment of these pathologies is of paramount importance to restore patients’ physical and mental well-being.

In some cases of severe corneal pathologies, a transplantation of a donor cornea is generally the only procedure to restore one’s vision without good alternatives. First performed successfully by Zirm in 1905, keratoplasty has become not only the most commonly performed, but also the most successful allograft surgery with the cornea’s avascular characteristic minimizing risks of rejection post-transplantation [31,71]. There are several types of corneal transplantations that can be grouped into either full-thickness penetrating keratoplasty (PK) and partial lamellar surgeries that only involve replacing part of the cornea. These partial lamellar surgeries can be further subdivided into anterior, such as superficial anterior lamellar keratoplasty (SALK) and deep anterior lamellar keratoplasty (DALK), and posterior, such as Descemet stripping automated endothelial keratoplasty (DSAEK) and Descemet membrane endothelial keratoplasty (DMEK) [71]. The need for corneal transplants arises from failure or irreversible damage to any layer of the cornea. In decreasing order of frequency, the three most common entities needing corneal transplants are endothelial dystrophy, pseudophakic corneal edema, and corneal ectasia and thinning (e.g., keratoconus) [72]. The specific procedure thus depends on the specific pathology. For example, diseases affecting only the endothelium are preferably treated with DSAEK or DMEK rather than PK due to its less invasive nature [64]. Alternatively, patients suffering from isolated anterior stromal opacity are ideally treated with SALK [71]. Another procedure, keratoprosthesis, consists of completely replacing the natural cornea with an artificial one. This option is usually reserved for patients who experienced repeated corneal transplant failures or when poor outcomes are predicted with PK, such as for those suffering from Steven–Johnson syndrome [73,74].

### 3.4. Bioengineered Corneas: Addressing the Donor Shortage

Despite being a widely used treatment by corneal and anterior segment surgeons for irreversible and severe corneal conditions, the global shortage of suitable donor corneas remains a critical challenge. Currently, fewer than 2% of patients in need of a transplant will receive one, with the number of patients on the waitlist outnumbering available donors by a staggering 70:1 ratio worldwide. This imbalance is especially severe in countries with rapidly growing populations, such as India, China, and parts of Africa. Without the swift emergence of new treatments and advancements for corneal-blinding disorders—of which corneal transplantation is only the final step—this disparity is likely to worsen [2]. These statistics highlight the importance of developing alternative solutions. In such situations, bioengineered corneal tissues have emerged as the ideal avenue for future research and development.

## 4. Properties Relevant to Corneal Tissue Engineering

### 4.1. Key Properties to Reproduce

Among the vast extent of suitable materials for hydrogel synthesis, researchers should select the substrates that allow the hydrogel to most closely imitate the natural tissue it is replacing. In the case of corneas, the key properties that must be considered are biocompatibility, transparency, mechanical strength, and permeability [75]. A foreign object placed into the body should neither cause any direct damage nor illicit any strong immune response against the inserted material. Hence, it is crucial to ensure that both the substrate and any of its degradation products are non-toxic as well as non-immunogenic [75]. Considering the cornea’s significant contribution to the eye’s total refractive power, its transparency is a paramount characteristic that must be achieved by engineered tissues. Considering the cornea’s function of light transmission, it is essential to utilize materials capable of maintaining high transparency levels to ensure the patient’s high visual quality. Spectrophotometry demonstrated that in the visible light range of the electromagnetic spectrum, the cornea’s transmittance ranges from 80% for blue light with gradual increases to just over 90% on the red end [36]. The transmittance rapidly decreases at both extremities, as the anterior corneal layers block ultraviolet (UV) radiation to protect the inner eye while water molecules absorb wavelengths in the infrared region [76,77]. On the subject of light transmission, another optical characteristic to recreate is the natural cornea’s refractive index of 1.376 [78]. As for mechanical properties, the hydrogel must possess enough strength to not only to maintain optimal functionality once implanted, but also to withstand stretching and folding during its insertion [79]. The elastic modulus is one important mechanical property that needs to be taken into consideration when fabricating hydrogels [80]. This property indicates the degree to which an object can resist deformation when a force is applied onto it. In other words, the elastic modulus represents how easily a material can be stretched. In the case of the cornea, the elastic moduli of all layers range from less than 1 kPa to around 100 kPa, as listed in Table 1 [81]. Table 1 also contains other relevant properties for corneal tissue engineering separated by layer. Hydrogel stiffness is an important property due to its ability to regulate cell proliferation responses. An investigation by Gouveia et al. (2019) proposed that more compliant substrates promoted LESC division, while stiffer ones rather supported their differentiation [82]. Corneal tissue must also maintain good permeability since it mainly receives nutrients and other important molecules from diffusion from the aqueous humor. Experimentally, the diffusion coefficients were found to be approximately 2.6 × 10^−6^ cm^2^/s for glucose and 1.0 × 10^−7^ cm^2^/s for albumin [83]. It is thus crucial to choose the scaffold material that offers properties as close to the native cornea as possible to ensure optimal anatomical and functional outcomes.

### 4.2. Hydrogel Materials for Corneal Tissue Engineering

Potential polymers used for corneal tissue engineering can be derived from various origins, with each type bringing along its unique characteristics. Among natural polymers, collagen is a first option to consider due to its naturally abundant state in the cornea [36]. It therefore has optimal biocompatibility, biodegradability, and transparency, as well as an innate ability to interact with biological components [85]. Collagen’s main drawback is its mechanical properties that have been frequently deemed insufficient to withstand surgical operations [86]. Gelatin is an equally biocompatible alternative, but possessing greater flexibility and cost-effectiveness. Similarly to collagen, it also has poor mechanical strength [87]. Hyaluronic acid’s main advantage is its biocompatibility, but its mechanical strength is far from sufficient and it tends to dissolve in liquid environments [6]. Compared to the previous options, chitosan has better mechanical strength, anti-microbial properties, and good permeability to essential molecules. However, its brittleness illustrated by its tendency for breakage during surgeries remains a challenge to overcome [88].

Synthetic polymers offer unique advantages, especially in terms of reproducibility and customizability [75]. Poly-lactic-co-glutamic-acid (PLGA) is a polymer whose strength, pH, and swelling capacity can be modulated by changing the proportions of its constituents. This ability has gathered attention from researchers with the goal of finding the ideal ratio that creates a gel most closely resembling the native cornea [89]. PEG, already widely used in tissue engineering due to its adaptability, high transparency, and water retention ability, is another suitable material for corneal scaffolds [90]. Its good biocompatibility also facilitates the survival of corneal keratocytes and endothelial cells [91]. Polyvinylidene fluoride (PVDF) has satisfactory biocompatibility and high mechanical strength, but this material is underexplored, with few in vivo studies on PVDF to date [75]. While generally having better mechanical properties compared to its natural counterparts, the absence of cellular recognition signals is where synthetic polymers fall short. This may prevent the implant from completely integrating with the surrounding human tissues [75]. To address this shortcoming, natural polymers can be mixed with synthetic ones to combine their strengths while reducing their individual weaknesses. Gelatin reacting with methacrylic anhydride creates methacryloyl gelatin (GelMA), a strong gel that has been shown to support endothelial cell growth with good viability despite its high production costs [92]. The combination of PEG and chitosan possesses adequate attributes in terms of biodegradability, mechanical strength, and transparency, but is still underdeveloped [93]. Table 2 summarizes the strengths and limitations of each type of polymer for corneal tissue engineering. Thus far, hybrid materials show great promise, but with few in vivo studies, further investigations are required to evaluate their physical and biological qualities more extensively [32].

### 4.3. Stimuli-Responsive Hydrogels

Although hydrogels already possess base properties that can be adjusted by changing the material, newer models have also been capable of responding to environmental stimuli. Known as smart hydrogels, they can be fabricated to react to physical, chemical, or biological stimuli [4]. Physical triggers include light, temperature, electromagnetic field, sound waves, and pressure. Chemical stimuli can be pH, ion concentration, or redox conditions. Biological triggers consist of the presence of enzymes or other organic compounds like glucose [4,95,96]. Multi-stimuli-responsive hydrogels that can react to a combinations of different stimuli are also under development, as this feature enables them to be adapted to the human body’s complex internal conditions and environments [97].

Such smart hydrogels have been identified as ideal drug delivery systems able to react to specific stimuli that trigger the release of active ingredients [98]. Their ability to precisely regulate drug distribution leads to improvements in efficacy while minimizing harmful drug side effects [99]. These gels each have unique mechanisms of action depending on the stimulus they are sensitive to. For instance, with pH-responsive gels, variations in pH alter the ionization states of its acidic or basic functional groups, resulting in changes in its swelling capacity [100]. Guar gum succinate, for instance, contains acidic functional groups that would ionize and induce swelling at the intestinal pH of 7.4 [101]. Azobenzene, a photosensitive material, undergoes trans–cis isomerization when irradiated with light of a specific wavelength [102]. Table 3 provides a more extensive description of different smart hydrogels. These innovative biomaterials have prompted researchers to explore their potential applications with one such possibility being in the realm of cancer treatments. Knowing that cancer cells create microenvironments with pH levels lower than those of normal tissue, pH-sensitive gels have been proposed as drug carriers aimed at releasing chemotherapy agents specifically at the tumor site [103,104].

Smart hydrogels boast advantageous properties allowing for versatile applications across numerous fields. More specifically for corneal tissue repair, one of the most relevant characteristics is thermo-responsiveness [117]. While liquid at room temperature, it can turn into gel form when it reaches body temperature. Potential applications being explored include drug delivery and wound healing, considering its ability to penetrate into deeper regions of the eye or to mold into the shape of the injury site while it is still in liquid form [117,118]. As an example, Liu at al. (2019) have developed temperature-sensitive hydrogels as drug delivery systems to treat corneal neovascularization. At room temperature, the medications would dissolve into the liquid solution, but once in the eye, the polymers would gelate and release the active molecules sustainably over 1 month [119].

## 5. Role of Hydrogels in Corneal Tissue Engineering

### 5.1. Hydrogel Synthesis Processes

There are numerous processes to prepare hydrogels, with the main methods being physical and chemical crosslinking. Physical crosslinking involves polymer gelating via the formation of reversible intermolecular interactions (ionic bonds, H-bonds, etc.) without the need of external crosslinking agents [10]. In consequence, these gels are easily degradable, so they are generally unsuitable for long-term applications [120]. Physical crystallite formation is one specific method to induce physical crosslinking, and it consists of multiple cycles of freezing the polymer solution to −15 °C, then quickly melting it at room temperature until it gelates [121]. Each cycle of freezing and thawing draws the polymers closer together by forming more and stronger bonds [121]. 

In contrast to physical crosslinking techniques, chemical crosslinking involves the formation of intramolecular interactions (covalent bonds) that grant the product greater thermal, chemical, and mechanical stability [94]. Chemical crosslinking can be induced via a variety of techniques, including addition of small molecule crosslinkers, radiation crosslinking, and enzymatic crosslinking. Mono- and bi-functional molecules such as formaldehyde and glutaraldehyde have been extensively reported as powerful crosslinking agents [10]. These small molecules contain lone pairs that would react with the outward-facing, positively charged functional groups on each polymer, thereby linking them with covalent bonds [122]. The main downside of this method is the toxicity of the crosslinkers that negatively affect the end product’s biocompatibility [120]. Radiation crosslinking is a procedure that involves using high-energy electromagnetic waves to create free radicals that would initiate the polymerization process [123]. Unfortunately, the required equipment for radiation crosslinking is expensive and limits any current attempts for large-scale implantations [123]. Enzymatic crosslinking, a significantly cheaper alternative, uses catalysts like tyrosinase, horseradish peroxidase, or transglutamases [124]. These enzymes would catalyze the formation of covalent bonds between polymer chains, offering fast gelation speeds with high controllability [125,126]. Radiation and enzymatic crosslinking have the advantage of avoiding the usage of harmful chemicals [120].

### 5.2. Cell Encapsulation and Bioactive Molecule Integration

For cell encapsulation, multiple strategies have been identified, with the main goal being to create optimal 3D polymer networks that best ensure cell viability. Out of these strategies, 3D bioprinting has been at the forefront of developments in bioengineering [127]. Bioprinting corneas has been a subject of great interest, and to do so, researchers would integrate the desired cells into the bioink, and the matrix would be printed with cells already embedded within it [128]. The exact formulation would depend on the specific technique. On the one hand, lower cell density is preferred when using inkjet printing since higher viscosities risk clogging the nozzle [129]. On the other hand, extrusion-based printing favors high cell density since it functions better when using high-viscosity substances [130]. The smaller the nozzle, the more aligned and structured the printed ECM becomes, but the squeezing of the bioink through a tighter nozzle increases the risks of cells being damaged by shear forces [128]. Laser-based bioprinting is nozzle-free and thus avoids this issue [131]. However, it comes with its own limitations related to cells dying from thermal damage if the laser power is set too high [130]. Electrospinning has also emerged in recent years as a technology capable of producing 3D nanofiber scaffolds. This innovation uses electrostatic repulsion forces generated by a high-voltage power supply to eject the polymer solution through a needle tip and onto a collector plate [132]. In this case, the main challenge is the preservation of adequate cell activity during this process since the electrostatic forces separating positively and negatively charged groups may distort the specific polymer arrangement required for cell survival [133]. Further research is necessary to find solutions that can address the limitations of each technique.

Taking advantage of most hydrogels’ high water absorption capacity and porosity, bioactive molecules can easily be integrated inside these structures. It is even possible to control the speed of bioactive molecule release by selecting different polymers that, in turn, adjust the gel’s swelling capacity, rate of degradation, and other mechanical properties [75]. In one experiment, fibroblast growth factors inserted into a gelatin-based structure demonstrated satisfactory drug-eluting kinetics for up to 20 days, enhancing corneal cell survival [87]. Another approach to drug delivery under development is the utilization of surface modifications. Free radicals or functional groups can be added to the hydrogel with one end bonding to its surface and the other end attached to an active ingredient [134]. This strategy works best in cases where cells only need to be seeded on the surface of the scaffold, such as for the endothelium, with the molecules having difficulty penetrating deeper inside [135].

## 6. Clinical Applications of Hydrogel for Corneal Defects

### 6.1. Hydrogels for Corneal Pathologies

In practical terms, hydrogel-based corneal tissue possesses numerous potential applications ranging from single-layer repair to full-thickness replacement [136]. Considering the wide array of potential materials and synthesis techniques, different teams have devised various fabrication processes according to the desired properties and pathology to treat. In recent years, hydrogel-based scaffolds have been tentatively used to treat corneal perforation as an alternative to PK [137]. N-butyl cyanoacrylate tissue adhesives (CTA) and human fibrin glue (HFG), due to their good biocompatibility, are among the most commonly described materials used for such cases [138]. In 73 cases of fungal keratitis-induced corneal thinning or perforation treated with CTA by Garg et al. (2003), resolution with scar formation was seen in 64% of eyes, saving them from needing surgical interventions [139]. It should be noted that over half of the successfully treated cases required multiple applications of the adhesive, suggesting that its healing effects are short-lived and that repeated usages are necessary until complete resolution [139]. In another series of similarly managed perforations associated with herpetic keratitis, Moorthy et al. (2010) reported a significantly lower cure rate of 37%, potentially due to the recurrent nature of that infection [140]. Siatiri et al. (2008) found an 83% cure rate using HFG to repair corneal perforations of both infectious and non-infectious etiologies [141]. This higher success rate may, however, be attributed to the inclusion of mostly small-sized lesions in that study, with experts generally agreeing that fibrin and cyanoacrylate are equally effective for perforations under 2 mm [141,142]. Other trials have explored PEG-based hydrogels as bandages to promote corneal incision closure post-operative. These studies concluded with similar findings, where the hydrogel sealant facilitated wound closure, decreased astigmatism, and reduced patient discomfort [143,144]. In an attempt to utilize hydrogels beyond just wound healing, Islam et al. (2018) transplanted collagen and phosphorylcholine-based implants by anterior lamellar keratoplasty in unilaterally blind patients presenting high risks of allograft rejection. Over the course of two year-long follow-ups, patients experienced variable improvements in visual acuity, with considerable reduction in pain, irritation, or tearing caused by the underlying etiology [145]. Likewise, Buznyk et al. (2015) implanted similar scaffolds in another group of high-risk patients suffering from corneal ulceration, achieving significant symptom relief and visual acuity improvements in two patients out of three [146]. Certain studies have also examined the viability of responsive hydrogels to promote corneal wound healing. In particular, animal experiments performed by Xu et al. (2020) investigated a poloxamer and ε-polylysine-based thermosensitive gel as a drug delivery system that would undergo phase transition at around room temperature. When loaded with bone morphogenetic protein 4, their gel was capable of inhibiting corneal neovascularization during the corneal healing process [147].

Thus far, clinical trials have presented hydrogels as not only safe, but also effective for certain diseases. Notably, a significant advantage of these fabricated corneas is that they frequently do not require patients to be on prolonged immunosuppression regimes, as opposed to recipients of human corneas [148]. When appropriate polymers are used, these hydrogels can even promote nerve regeneration as well as stromal cell repopulation [145]. While these results are promising, the number of studies remains small due to the novelty of this innovation. Even among those that exist, most trials incorporated small numbers of participants and mainly focused on fibrin, collagen, or PEG despite the diversity of materials, therefore offering limited knowledge into hydrogels’ full potential [58]. As a step in the right direction, researchers have begun testing wider varieties of hybrid materials, starting with preclinical animal studies [149]. Selecting the ideal substrate and the most suitable synthesis technique remains a challenge meriting further research [58]. Furthermore, currently available data suggest that hydrogels are most effective for small incisions, with success rates plummeting for any perforation bigger than 3 mm in diameter, suggesting that improvements to their effectiveness need to be made [58].

### 6.2. Hydrogels for Corneal Tissue Engineering

While there exist numerous corneal applications of hydrogels, those specifically designed for corneal tissue engineering remain in an early phase of development, with the latest investigations on this topic being at the preclinical stage. One study by Agrawal et al. (2024), consisting of both in vitro and in vivo components, evaluated the viability of hydrogels as regenerative treatments for corneal defects [150]. Their model, made from photo-crosslinked hyaluronic acid and gelatin called Kuragel, not only displayed transparency and compressive strengths comparable to human corneas, but also provided a suitable environment for corneal stromal cells. In fact, the in vitro testing resulted in a cell viability of around 95% after 4 weeks [150]. When implanted in injured rabbit eyes and observed over a 90-day period, in the treated group, corneal clarity restauration and complete re-epithelization occurred within one month without complications. By the end of the assessment period, imaging also revealed healthy stratified stromal tissue as well as extensive nerve regeneration [150]. In another study, Kang and colleagues (2024) fabricated an in situ collagen and hyaluronic acid-based hydrogel enhanced with growth factors, and investigated its effectiveness for corneal repair [151]. Upon gelation, the hydrogel demonstrated adequate mechanical and biological properties. To evaluate its cytocompatibility, Kang’s team seeded corneal endothelial cells in the scaffold and observed cell proliferation at a near 100% viability rate [151]. In their in vivo rat models, treated corneas exhibited a complete recovery of corneal thickness with minimal scarring and no epithelial hyperplasia in the short span of 7 days [151]. Shen et al. (2023) designed unique gels by mixing porcine decellularized corneal stromal matrix (p-DCSM) with methacrylated hyaluronic acid (HAMA) and tested samples made with varying p-DCSM–HAMA ratios (1:1, 1:2, 3:2, 3:1) [152]. On top of combining p-DCSM’s biocompatibility with HAMA’s structural stability, all models promoted epithelium, stroma, and nerve regeneration in rabbit eyes during the 2-month follow-up period, with the 3:1 combination achieving the best results [152]. Porcine corneal stroma is less restricted by donor sources, but if the decellularization process is imperfect, leftover xenoantigens in the tissue may induce immune rejection [75]. To overcome this barrier, future avenues first involve improving decellularization procedures before moving further forward with xenografts [153]. GelMA, PEG diacrylate, Pluronic F127, type I collagen, acrylated gelatin, and thiolated gelatin are examples of other materials under investigation that may be suitable for corneal regeneration [149,154,155].

So far, studies on corneal tissue engineering produced satisfactory results, but this technology is still in its early days. With good outcomes in animal models, researchers should consider turning their attention towards implementing these mimetic scaffolds in human patients, with the ultimate objective of alleviating the dependency on donated corneas. To date, the survival rate of low-risk PK exceeds 90% at 5 years but drops below 70% in higher-risk recipients [156]. Through continuous advancements, these values represent the standard that bioengineered tissue should strive to achieve, if not surpass.

## 7. Challenges and Future Directions

Even though current results show promise, much more needs to be done prior to implementing hydrogel-based treatments at a larger scale. Notably, the extensive array of possible biomaterials and fabrication procedures for hydrogels poses significant difficulties for its regulation. In the United States, the Food and Drug Administration (FDA) classifies hydrogels as implantable devices, which must undergo extensive scrutiny and attain high safety standards before their approval [157]. When active molecules are incorporated into the scaffold, it becomes a combination product that falls under multiple categories, thereby increasing the number of regulatory standards to meet. In such cases, the approval process often takes 7–10 years, significantly limiting the commercial viability of such products [158]. At the time of writing, over 100 hydrogels have been approved by the FDA, with just as many undergoing trials [159]. Notable examples of commercially available materials for such usages include PEG, silicone, PVA, carbomer copolymer type A, and PLGA [159]. Despite their availability, such products currently have restricted clinical usages due to their novelty as well as their limited effectiveness [58]. By combining the advantages of both natural and synthetic materials, hybrid gels also deserve additional attention. One unique study by Vijayan and Kiran (2023) evaluated hybrid collagen–dextran–gadolinium oxide gels and documented their gel’s excellent ability to promote corneal regeneration in vitro [160]. Such reports emphasize the importance of testing novel materials in order to continuously discover more effective combinations. With regard to ocular applications, most existing hydrogels are either administered as lenses or drug delivery systems. Very few have attempted to use hydrogels for corneal tissue engineering due to the cornea’s complex anatomy and physiology [159]. Existing studies have at most only explored partial-thickness hydrogel implants, illustrating another limitation to overcome [58]. Future explorations should be directed towards examining full-thickness scaffolds, with the end goal being to offer an alternative treatment to human corneal transplants for patients on a waitlist that will, more often than not, never reach their position.

With science advancing at rates faster than ever before, numerous novel trends have emerged in hydrogel research from its fabrication to its applications. Peptides have recently been proposed as a unique yet innovative material possessing not only the advantages of other natural polymers, but also functional sequences capable of integrating important biomolecules [161]. Emphasizing on their distinct levels of tunability, smart hydrogels have gathered more attention through the ever-increasing number of studies investigating and developing such products [4]. With ongoing trials examining intra-articular hydrogel injections, a similar route of administration can be considered for ocular usages, with its main strengths lying in its minimal invasiveness and its ability to mold into irregularly shaped sites [75,159]. For example, McTiernan’s team (2020) experimented with an injectable hydrogel made with PEG and fibrinogen with promising results [162]. Barroso and colleagues (2022) tested gelatin-based injectable adhesives capable of completely sealing small full-thickness perforations [163]. Although previously underdeveloped, smart hydrogels are also seeing a rise in popularity due to their sophisticated properties, meriting more extensive investigations [164]. While advancements are being made with regard to material selection, concurrent improvements in hydrogel synthesis technologies are also top priorities for bioengineers. Certain ongoing studies therefore aim to enhance 3D printing techniques in order to not only optimize the stability of the printed scaffold, but also to increase cell viability and proliferation [58]. Moving forward, subsequent steps would involve translating findings from fundamental studies into concrete clinical applications. The unprecedented customizability of hydrogels combined with the creativity of scientists brings about innumerable possibilities for groundbreaking innovations and personalized medicine.

## 8. Conclusions

This review highlights the principal discoveries in corneal tissue engineering, focusing on the use of hydrogel-based scaffolds. These materials have demonstrated promising outcomes in replicating key properties of the cornea, such as transparency, biocompatibility, and mechanical strength. Hybrid materials, combining natural and synthetic polymers, show enhanced potential, particularly in promoting corneal cell regeneration and maintaining transparency. However, the research is still in preclinical stages, with successful results mainly achieved in animal models. Stimuli-responsive hydrogels offer further innovation, allowing for controlled drug release and tailored treatment approaches.

To propel corneal tissue engineering forward, future research should prioritize full-thickness scaffolds and human trials. Collaborative efforts between materials scientists and clinicians are crucial to refine hydrogel formulations, improve synthesis techniques, and ensure regulatory compliance. These advancements could lead to the development of commercially viable, bioengineered alternatives to human corneal transplants, significantly reducing the dependency on donor tissues.

## Figures and Tables

**Table 1:** Physical and optical properties of each corneal layer.

Layer	Thickness (μm)	Refractive Index	Elastic Modulus (kPa)
Epithelium	40–50	1.400	0.57
Bowman’s layer	8–15	1.380	109.8
Stroma	470–500	1.369	33.1
Descemet’s membrane	10–12	Not reported	50
Endothelium	4–6	1.373	4.1
References	[84]	[78]	[81]

**Table 2:** Strengths and limitations of types of materials used for corneal tissue engineering.

Source	Examples	Strengths	Limitations	References
Natural	Collagen Gelatin Hyaluronic acid Chitosan	Good transparency Biocompatible Biodegradable Cost-effective	Insufficient mechanical strength Fragile Low stability	[6,85,88,94]
Synthetic	PLGA PEG PVDF	Good mechanical strength Greater stability Greater water absorption Chemically inert	Lower biocompatibility No reports on biodegradation Greater risk of foreign body response	[13,32,75]
Hybrid	GelMA PEG + chitosan	Good mechanical strength Good transparency Biodegradable	No in vivo studies Underdeveloped Complex and more expensive production process	[83,92,93]

PLGA: poly-lactic-co-glutamic acid, PEG: polyethylene glycol, PDVF: polyvinylidene fluoride, GelMA: methacryloyl gelatin.

**Table 3:** Examples and mechanisms of action of smart hydrogels.

Stimuli	Examples	Mechanism of Action	References
pH	Guar gum succinate PEI PVA	pH variations induce changes in the acidic or basic functional groups’ ionization state, leading to swelling or shrinking.	[105,106]
Temperature	Poloxamer PNIPA Glycerophosphate	Temperature changes disrupt the equilibrium state between hydrophobic segments, hydrophilic segments, and water, therefore inducing sol-gel transformations.	[107,108]
Light	Azobenzene Black phosphorus o-nitrobenzyl ester	Photosensitive functional groups (chromophores) undergo photoisomerization when exposed to visible or UV light, thus modifying the gel’s properties.	[109,110]
Electric field	Agarose Carbomer Calcium alginate	Electrostatic forces generated by the electric field induces positional changes and ion movements in charged polymer chains.	[111,112]
Enzyme	Hyaluronidase Cinnamyloxy groups	The presence of enzymes will degrade its corresponding specific linkage, causing morphological changes that subsequently modify the hydrogel’s physical properties and activity level.	[113,114]
Glucose	Concanavalin A Phenylboronic acid	Hydrogels containing these substances can detect and react to blood glucose levels, of which higher concentrations would increase the binding of glucose to the hydrogel. This would induce conformational changes to stimulate insulin secretion.	[115,116]

PEI: polyacrylamide, PNIPA: poly-N-isopropylacrylamide.

## Data Availability

No new data were created or analyzed in this study. Data sharing is not applicable to this article.
